# A comparative investigation of catecholamines and glucocorticoids impact on glioblastoma invasive behavior via 2D and 3D cell culture

**DOI:** 10.1371/journal.pone.0339764

**Published:** 2026-02-11

**Authors:** Yasaman Moazen Safaei, Reza Mahdavian, Hossein Soleymani, Negar Rahimpour, Abdollah Allahverdi, Hossein Naderi-Manesh

**Affiliations:** 1 Department of Biophysics, Faculty of Biological Sciences, Tarbiat Modares University, Tehran, Iran; 2 Department of Stem Cells Technology and Tissue Regeneration, Faculty of Interdisciplinary Science and Technology, Tarbiat Modares University, Tehran, Iran; University of Molise Department of Medicine and Health Sciences “Vincenzo Tiberio”: Universita degli Studi del Molise Dipartimento di Medicina e Scienze della Salute Vincenzo Tiberio, ITALY

## Abstract

Prolonged or intense stress can contribute to the progression of various pathological conditions, including cancer. To explore how stress affects glioblastoma invasiveness, U87-MG cells with reduced proliferation were treated for four days with epinephrine or hydrocortisone (low/high doses) in 2D and 3D cultures. Migration, cell stiffness, and vimentin expression were assessed. In 2D scratch assays, the scratch closure rate was 46.4% in the control group. Treatment with epinephrine increased this rate up to 97.0%, while hydrocortisone reduced it to 13.3%. In 3D cultures, both treatments inhibited spheroid elongation; however, only epinephrine significantly enhanced cell dispersion. Compared to the control group (9.00 kPa), mean stiffness decreased down to 1.92 kPa with epinephrine and increased up to 26.28 kPa with hydrocortisone. Vimentin expression was significantly upregulated under all treatment conditions. Overall, epinephrine promotes glioblastoma invasiveness, while hydrocortisone limits migration. Although cell stiffness changes align with migratory results, vimentin is possibly involved in different mechanisms affected by these compounds.

## Introduction

The term ‘stress’ was originally introduced into medical terminology from physics. In physics, stress refers to the interaction between an applied force and a material’s resistance to deformation. Building on this definition, Hans Selye, who is often regarded as a pioneer in the field, conceptualized stress as a nonspecific body response to various demands, ranging from microbial challenges to emotional stimuli [1]. Several decades later, the stress response can still be simplified as the activation of neuroendocrine axes in reaction to a perceived threat [2]. However, this is an adaptive allostatic response; prolonged or intense exposure to stressors can ultimately lead to or exacerbate pathological conditions [3,4]. Therefore, understanding how stress response becomes maladaptive is crucial for uncovering the mechanisms underlying disease etiology.

Stress response has been proposed as a significant environmental factor contributing to cancer development [5,6]. The primary hypothesis linking stress and cancer is based on longitudinal [7] and retrospective [8] studies, which have yielded controversial results [9,10]. However, the lack of causal evidence in these approaches necessitates the use of experimental models to further investigate this interaction. To date, numerous studies have reported various mechanisms by which the stress response accelerates cancer progression [11–13] or contributes to its initiation [14,15].

One proposed explanation is that catecholamines [16–18] and glucocorticoids [19,20], which are key molecular modulators of the stress response, can directly influence the invasive behavior of cancerous cells. However, despite extensive research, there is still no consensus on whether these stress-related molecular modulators consistently suppress or enhance the invasiveness of cancer cells. Much of the current literature attributes this variability to factors such as cancer type [21], concentration-dependency [22], treatment duration, and other experimental design parameters [23].

To acquire migratory and invasive properties, cancer cells undergo a process known as epithelial-to-mesenchymal transition (EMT). During this transition, cells exchange epithelial characteristics for mesenchymal traits, leading to changes in morphology, polarity, adhesion, and motility. While molecular markers are commonly used to track EMT, a combinatorial approach is recommended for more precise monitoring [24]. Simultaneously assessing cell behavior, molecular alterations, and mechanical properties [25] provides a more comprehensive understanding of whether catecholamines or glucocorticoids influence EMT and, consequently, cancer cell invasion.

Glioma is an umbrella term encompassing neuroepithelial tumors of glial origin. Among its subtypes, glioblastoma is the most common and aggressive. The poor prognosis associated with glioblastoma is largely attributed to the extensive infiltration of cancerous cells into surrounding brain regions [26]. The potential role of stress in accelerating this invasive process has been explored in various *in vivo* and *in vitro* models [27–29].

This study aimed to develop an *in vitro* model to investigate how exposure to epinephrine (a catecholamine) and hydrocortisone (a glucocorticoid) affects the migratory behavior of the U-87 MG glioma cell line, which is likely of glioblastoma origin. Epinephrine and hydrocortisone were used to represent the Sympathetic-Adrenal-Medullary (SAM) and Hypothalamic-Pituitary-Adrenal (HPA) axes, respectively [2]. The activation of these systems is influenced by the type, intensity, and duration of the stressor. The U-87 MG cell line was selected for its controversial stress responsiveness and well-documented intrinsic invasiveness [30].

The study specifically aims to address the following questions:

- Does epinephrine or hydrocortisone, at physiological concentration ranges, alter the invasive behavior of U-87 MG cells?
- Is concentration a key factor influencing dual effects?

To provide comprehensive insights, both 2D and 3D cell culture models were employed. Mitomycin C was used to suppress the cell cycle, allowing for a specific focus on invasion rather than proliferation. Cell migration and invasive behavior were assessed by monitoring 2D cell layers and 3D spheroids using microscopy. To further characterize the potential for cell motility, intrinsic cell stiffness was measured as an indicator of migratory capacity. Additionally, Vimentin expression, a key EMT marker, was analyzed using flow cytometry.

The comet assay confirmed that short-term, low-dose mitomycin C treatment did not induce significant DNA damage. Flow cytometry further validated cell cycle arrest at the G2/M transition. Microscopic analysis revealed that epinephrine enhances the invasive behavior of U-87 MG glioma cells, whereas hydrocortisone suppresses it. Additionally, epinephrine treatment reduced cell stiffness, correlating with increased migration, while increased stiffness under hydrocortisone treatment was associated with suppressed migration. These trends were consistent across both 2D and 3D culture models. In line with microscopic observations, vimentin expression increased with epinephrine treatment. However, this consistency was not observed with hydrocortisone. The rise in this marker under hydrocortisone treatment may be linked to the induction of cancer stemness.

## Materials and methods

### Cell culture conditions and treatments

U-87 MG cells (Pasteur Institute, NCBI Code: C531) were cultured in DMEM (Gibco™ 12100061) supplemented with 10% Fetal Bovine Serum (FBS; Bioidea BI-1201) and 1% Penicillin-Streptomycin (Pen-Strep; Bioidea BI-1203). For cell detachment during passages, Phosphate-Buffered Saline (PBS; 1X, pH 7.4) and Trypsin-EDTA 1X (0.05%; Bioidea BI-1601) were used. The cells were incubated at 37 °C with 5% CO₂ and >95% humidity unless otherwise stated. Cells -were cryopreserved in 95% complete DMEM and 5% Dimethyl Sulfoxide (DMSO; Sigma-Aldrich, Cat. No. D2438) at −80°C and subsequently transferred to a liquid nitrogen tank (−196°C) for long-term storage.

### Spheroid formation

Spheroids were generated using the hanging drop method. 20 µL drops of cell suspension, each containing 50000 cells, were placed as hanging drops on the lid of non-adherent culture plates. To prevent evaporation, the surface of the plates was covered with three mL of sterilized PBS. After 48 hours, spheroids formed and were monitored under a stereo zoom microscope (Labomed CZM6). Compact spheroids were harvested by adding fresh media to the drops.

All treatments were performed under aseptic conditions at 37°C.

### Mitomycin C

#### Stock preparation.

Each vial of mitomycin C (Mitonco, Korea United Pharm) contains 2 mg of the drug. To prepare the stock solution, 5 mL of sterilized PBS was added to the vial, and the solution was stored in the dark at 4°C for up to two weeks.

#### Treatment protocol.

For 2D cell cultures, mitomycin C was administered at a concentration of 5 µg/mL for 2 hours. In 3D cultures, the treatment duration was extended to 3 hours. Following treatment, the cells were washed twice with sterile PBS.

### Epinephrine

#### Stock preparation.

A 5 mM stock solution of epinephrine (Iran Hormone Company) was prepared and subsequently diluted in DMEM to achieve an intermediate concentration of 5 µM. Concentrations of 2 µM and 200 nM were then used for cell treatment, based on reported physiological levels.

### Hydrocortisone

#### Stock preparation.

0.005 g of the active ingredient hydrocortisone (Sina Daroo Company) was dissolved in 1 mL of DMSO to prepare a 10 mM stock solution. A fresh stock solution was prepared for each treatment. Intermediate concentrations (1 mM to 10 µM) were prepared in DMEM, and final concentrations of 5 µM and 500 nM were used, based on physiological levels reported in the literature.

Cells were treated with epinephrine or hydrocortisone for four days, with half of the medium replaced with fresh treatment-containing medium every other day. Throughout the treatment period, the FBS concentration was maintained at 5%.

### Assays

#### Comet assay after low-dose, short-term mitomycin C treatment.

To control DNA damage induced by mitomycin C treatment, cells were exposed for two hours to one of three conditions: 5 µg/mL mitomycin C, 5% H₂O₂ (positive control), or untreated media (negative control). Following treatment, the cells were washed twice with sterile PBS to eliminate any residual compounds. The comet assay was performed according to the referenced protocol [31], with minor modifications. To evaluate DNA damage, plasma-treated glass slides (HARRICK PLASMA, PDC-FMG-2 and PDC-32G-2) were initially coated with 1.5% agarose (Merck, A6877) dissolved in PBS. Harvested cells were suspended in 0.8% low electroendosmosis agarose (Fermentas, R0499) prepared in TAE buffer (Tris-Acetate-EDTA) and layered onto the slides. The samples were then sequentially incubated in lysis buffer, PBS, and denaturation buffer, followed by electrophoresis in TBE buffer for 15 minutes. After staining with ethidium bromide, DNA damage was assessed using fluorescence microscopy (Olympus IX81).

#### Cell cycle flow cytometry after low-dose, short-term Mitomycin C treatment.

To confirm cell cycle arrest, both mitomycin C–treated and control cells were analyzed using flow cytometry. Cell suspensions (10⁴ cells per sample) were fixed by adding the sample dropwise to 3 mL of 75% ethanol (–20°C) while vortexing. The samples were then stored at –20°C for 2 hours before being centrifuged at 200 × *g* for 10 minutes.

The resulting pellets were resuspended in 1 mL of cold PBS at 4°C. After washing with PBS, 2 × 10⁴ cells were added to 200 µL of the staining solution. The staining solution (10 mL total) consisted of 10 µL Triton X-100 (final concentration: 0.1% v/v), 5 mg RNase A (Sigma-Aldrich, R5503), and 0.4 mL of a 500 µg/mL propidium iodide (PI) solution. RNase A (10 mg in 2 mL distilled water) was boiled for 5 minutes to eliminate DNA contamination. The samples were then incubated at room temperature for 30 minutes before being analyzed by flow cytometry.

#### Cell migration assay for 2D and 3D cell culture.

To investigate whether epinephrine or hydrocortisone affect the invasive potential of glioblastoma cells, migration assays were performed using U87-MG cells. The cells were treated with epinephrine at concentrations of 2 µM and 200 nM, or hydrocortisone at 5 µM and 500 nM. Untreated cells served as the control group.

In 2D cultures, cell migration was assessed using a scratch assay. Once the cells reached 90–100% confluency, mitomycin C was applied to inhibit proliferation prior to scratching. A scratch was then made using a 1 mL pipette tip. Images were captured every 24 hours over a four-day period using a light microscope (Leica, LEITZ DM IL). Scratch closure was calculated using the formula: ((Area at 0 h – Area at x h) × 100)/ (Area at 0 h), based on the measured scratch areas using ImageJ at each time point.

In 3D cultures, cells were initially treated with mitomycin C, washed, and subsequently exposed to either epinephrine or hydrocortisone. Spheroid deformation and dispersion were monitored using light microscopy over a four-day period. To quantitatively assess changes in spheroid shape, several morphological features were extracted using ImageJ. Circularity was calculated as 4π × Area/ Perimeter² and provided a measure of how closely the spheroid shape resembled a circle. The aspect ratio, defined as the ratio of the major axis to the minor axis, indicated the degree of elongation. Roundness, calculated as 4 × Area/ (π × Major axis²), reflected overall shape distortion. Solidity, defined as the ratio of the spheroid area to its convex hull area, described how compact or irregular the spheroid was.

#### Measuring cellular stiffness with bio-AFM in 2D and 3D culture models.

To assess whether epinephrine or hydrocortisone affects the mechanical properties of glioblastoma cells, bio-AFM (atomic force microscopy) was used. This technique enabled precise measurements of cell stiffness following treatment and has been successfully used in our laboratory [32]. Five conditions were tested in both 2D and 3D cultures: epinephrine at 200 nM and 2 µM, hydrocortisone at 500 nM and 5 µM, and an untreated control. For 2D cell culture, the cells were seeded at a density of 1.2 × 10^5^ cells per well on plasma-treated coverslips and maintained in a humidified incubator at 37˚C and 5% CO2. After 4 days of treatment, the samples were washed with pre-warmed PBS (37˚C) and mounted on the bio-AFM (Nanosurf bio-AFM, CGS10/Au tip from NT/MDT company) set up. Bio-AFM measurements were performed in liquid mode using PBS at 37 °C. Moreover, for 3D culture of U87 MG cells, the spheroids were prepared on coverslips as described above, and a similar protocol was followed to assess their cellular stiffness. Force–distance curves were collected at an indentation rate of 2 µm/s, with maximum indentation depths of approximately 500 nm. A Poisson’s ratio of 0.5 was assumed for all calculations. The cantilever had a nominal spring constant of 0.02 N/m, and the deflection sensitivity (0.128 V/nm) was applied during data processing via the read-in calibration.

#### Evaluating the effect of epinephrine and hydrocortisone on EMT- markers expression.

To evaluate whether the treatments promote epithelial-to-mesenchymal transition (EMT), vimentin and CD44 expressions as key EMT markers [33] were measured using flow cytometry. Cells were treated for four days in 2D monolayer culture with two concentrations of epinephrine (200 nM and 2 µM), two concentrations of hydrocortisone (500 nM and 5 µM), or left untreated as a control. After treatment, cell suspensions (10⁵ cells per sample) were fixed in 4% paraformaldehyde for 15 minutes at 4 °C. Following centrifugation (at 200 × g for 5 minutes), the cells were washed once with PBS. For CD44 evaluation, at this step, samples were stained with APC anti-human CD44 antibody (50 µg/mL; BioLegend Cat. No. 338806), diluted in PBS with 5% FBS. However, for vimentin assays, fixed samples were subsequently permeabilized to allow antibody penetration. Permeabilization was performed by incubating the cells in 0.1% Triton X-100 (prepared in PBS with 5% FBS) for 15 minutes on a slow rocking shaker at 4°C. Following permeabilization, the cells were washed and centrifuged again as previously described. After the PBS washes, the cells were stained with an FITC-conjugated anti-human vimentin antibody (6 µg/mL; Padzaco, PDZMM115), diluted in PBS with 5% FBS. Staining was carried out for 20 minutes at 4°C in the dark on a rocking platform, and the samples were subsequently analyzed by flow cytometry.

In parallel, vimentin localization was assessed using fluorescence microscopy (Olympus IX81). Cells were seeded onto slides, washed twice with PBS, and fixed with 4% paraformaldehyde for 20 minutes at room temperature. Following two additional washes with PBS, the cells were permeabilized with 0.1% Triton X-100 for 15 minutes and then washed again. Staining was performed using a FITC-conjugated anti-vimentin antibody (6 µg/mL) and DAPI for 15 minutes at room temperature in the dark. After rinsing with PBS, the slides were imaged using a fluorescence microscope.It is noteworthy that vimentin assays were performed in triplicate as the main EMT marker. In contrast, CD44 expression was evaluated as an additional EMT marker in a single confirmatory experiment. Owing to the exploratory nature of this analysis, microscopy was not performed, and the results are presented in the Supplementary Information.

### Data analysis

Microscopic images were quantified using ImageJ (version 1.54f; Fiji). Young’s modulus was determined by analyzing bio-AFM data with AtomicJ (version 2.3.1). Flow cytometry data were analyzed using FlowJo (version 10.10.0). Statistical significance and graphs were assessed and generated using R (version 4.3.1).

## Results

### Culture conditions and pre-treatment validation

To ensure that the results were both comparable and broadly applicable, experiments were performed using both 2D and 3D U87-MG cell culture systems for microscopy-based migration analysis and bio-AFM measurements. Successful spheroid formation in the 3D model is shown in S1 Fig. To achieve a uniform cellular response and isolate the effects of treatment on invasion rather than proliferation, it was necessary to maintain the cells in a single generation. Therefore, mitomycin C was used to inhibit proliferation [34]. The absence of significant DNA damage following mitomycin C treatment was confirmed by comet assay (S2 Fig), and effective cell cycle arrest was validated by flow cytometry (S3 Fig), both of which are presented in the supplementary data.

### Epinephrine and hydrocortisone can modulate invasive behavior in U87-MG cells

To mimic the stress response, epinephrine and hydrocortisone were used at physiological concentrations of catecholamines [34] and glucocorticoids [35,36] typically found in the body fluids under both normal and stressful conditions. The concentration ranges for these two molecules were calibrated to facilitate meaningful comparisons, spanning from the low hundreds of nanomolar to approximately tenfold higher.

### Epinephrine promotes, while hydrocortisone inhibits, glioblastoma cell migration

**Fig 1:**
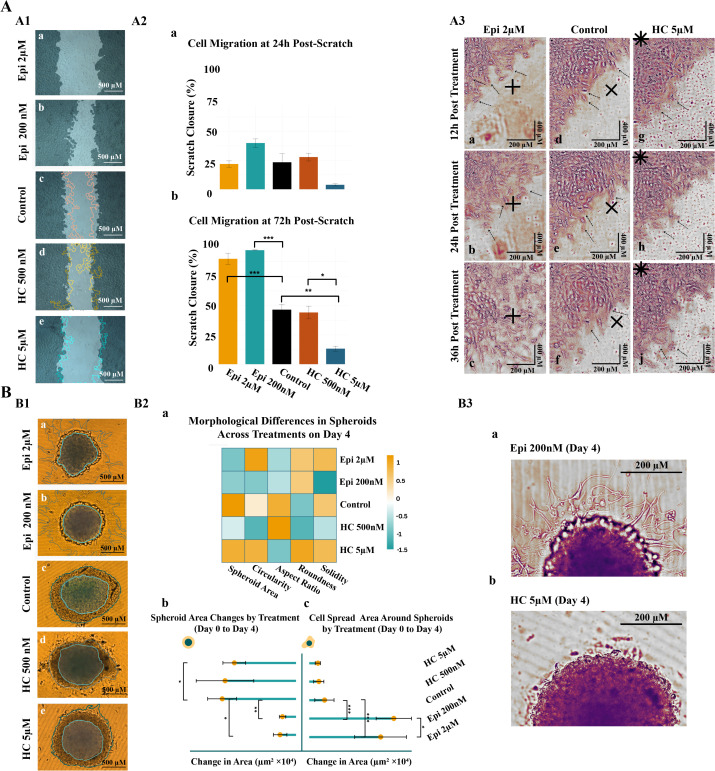
Assessment of cell migration in both 2D and 3D cell culture following treatments. Epi stands for epinephrine and HC stands for hydrocortisone. **(A)** 2D scratch assay. **A1:** Brightfield images of cell migration at different timepoints following treatment. The dark shaded region indicates the scratch area remaining at 24 hours post-treatment, and the lighter central area shows the remaining scratch at 72 hours. Colored lines highlight the scratch borders. **(a)** Epinephrine 2 μM **(b)** Epinephrine 200 nM, **(c)** Control, **(d)** Hydrocortisone 500 nM, **(e)** Hydrocortisone 5 μM.**A2:** Quantification of scratch closure (%) across different treatment groups over time. **(a)** Cell migration at 24h post-scratch **(b)** Cell migration at 72h post-scratch. **A3:** Snapshots of cell migration in Epi 2 µM (panels a-c), hydrocortisone 5 µM (panels d-f), and control (panels g-j) groups at 12-, 24-, and 36-hours post-treatment (Scale bar: 200 µm). Black marks indicate approximately the same area across time points within each treatment group. Selected individual cells are indicated by arrows to facilitate tracking over time. **(B)** Spheroid-based migration assay (Scale bar: 200 µm). **B1:** Microscopic observation of spheroid morphology following treatments. The central dark shadow represents spheroid morphology at day 1, while the lower image layer shows spheroids at day 4. Spheroids and cell spreads are outlined in blue for better visibility. **(a)** Epinephrine 2 μM **(b)** Epinephrine 200 nM, **(c)** Control, **(d)** Hydrocortisone 500 nM, **(e)** Hydrocortisone 5 μM. **B2:** Quantification of spheroid-based migration assay. **(a)** Heatmap illustrating morphological differences in spheroids across treatments on day 4. The color scale represents morphological parameters, with warmer colors indicating larger values (up to 1) and cooler colors representing smaller values (down to −1.5). Area was normalized based on control, with 1 equaling the area of the control spheroid at day 4 and 0 representing the spheroid area at day 0. For other parameters: Circularity where 1 represents a perfect circle; Aspect Ratio where 1 shows least spheroid elongation; Roundness where 1 indicates least shape deformation; Solidity where 1 indicates the most compact spheroid shape with fewer protrusions. Lollipop graphs illustrate area changes in both spheroids (b) and cell spread regions **(c)**. **B3:** Example images showing the effect of treatment on cell migration in 3D cell culture on day 4. **(a)** Cells migrate out of the spheroid following Epinephrine 200 nM treatment. **(b)** Hydrocortisone 5 µM treatment inhibits cell migration out of the spheroid. Statistical significance is indicated by asterisks (* for p < 0.05, ** for p < 0.01, and *** for p < 0.001).

Stress molecular modulators can directly influence the invasive behavior of cancerous cells [17,20,21,17,37,38]. To explore this effect on glioblastoma cells, U87-MG migratory behavior was observed in both 2D and 3D cultures following epinephrine or hydrocortisone treatment. Fig 1 presents an overview of U87-MG migratory behavior under epinephrine and hydrocortisone treatments.

Starting with epinephrine treatment at both concentrations enhanced scratch closure and cell migration compared to the control in 2D cultures (Fig 1A1a and 1A1b). Although the difference was not statistically significant at 24 hours (Fig 1A2a), a strong and significant effect emerged by 72 hours (p < 0.001; Fig 1A2b). In the 3D culture model, this finding was further confirmed by the greater spread of cells from spheroids following epinephrine treatment over four days (p < 0.001; Fig 1B1a, 1B1b, and 1B2c). Notably, spheroid morphology also responded to treatment. While control spheroids tended to elongate over time, epinephrine-treated spheroids maintained a rounded morphology (Fig 1B2a). Changes in spheroid area were statistically significant (p < 0.01 for 200 nM and p < 0.05 for 2 µM epinephrine; Fig 1B2b). Interestingly, while no significant differences were detected between the two epinephrine concentrations in 2D cultures, in 3D cultures, the 200 nM dose led to a stronger effect than that observed with the 2 µM dose (p < 0.05; Fig 1B2c and 1B3a).

Unlike epinephrine, which enhanced cell migration, hydrocortisone exhibited an inhibitory effect. Hydrocortisone at 5 µM concentrate suppressed cell migration in both 2D and 3D systems, whereas the 500 nM concentration had minimal to no effect (Fig 1A1d, 1A1e, 1B1d, and 1B1e). Hydrocortisone 5 µM significantly slowed scratch closure compared to both the control and hydrocortisone 500 nM-treated groups (p < 0.01 and p < 0.05, respectively; Fig 1A2b). This inhibitory trend was also observed in 3D cultures, where hydrocortisone 5 µM-treated spheroids showed significantly reduced cell spread (Fig 1B2c), and less elongation compared to controls (p < 0.05; Fig 1B2b and 1B3b).

### Epinephrine and hydrocortisone significantly alter cell stiffness in opposing directions

**Fig 2:**
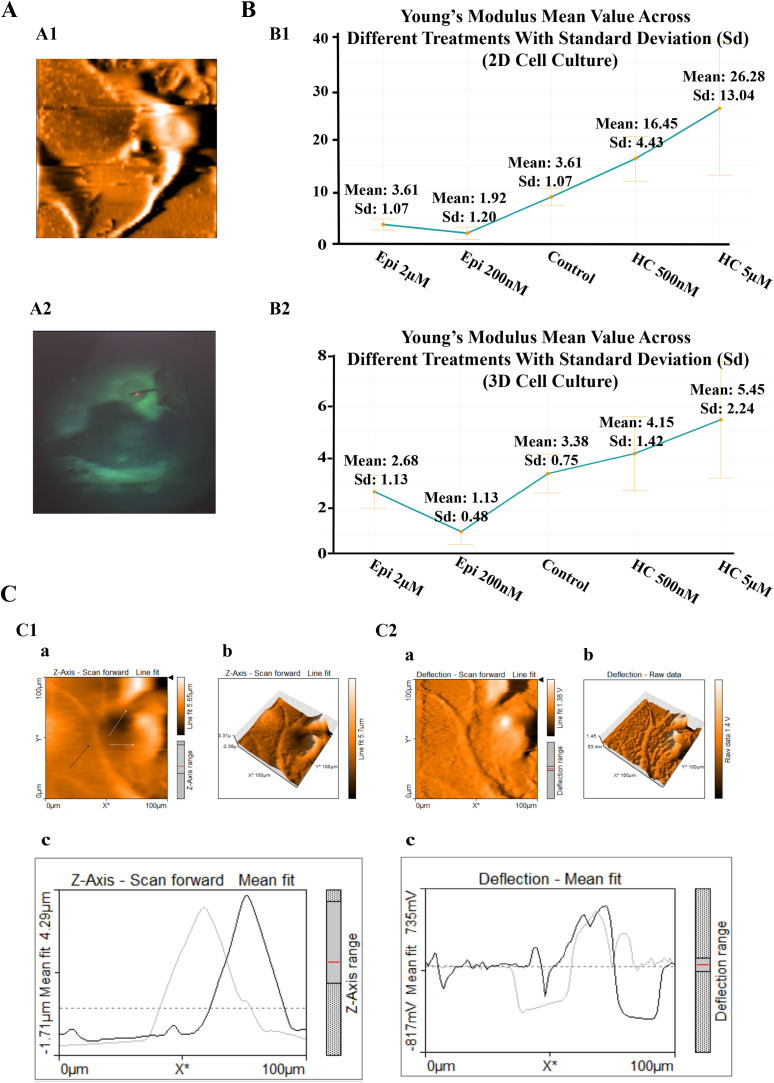
Bio-AFM analysis of 2D and 3D samples. **(A)** Sample Visualization for 2D and 3D Cultures. **A1:** Example cell surface topography obtained from forward deflection scanning by AFM (Scan area: 100 × 100 µm²). **A2:** Image of a spheroid positioned under the AFM tip; the red dot indicates the laser reflection point. **(B)** Average Young’s Modulus (kPa) values across treatment groups, with standard deviations shown. **B1:** Changes in average Young’s modulus (kPa) observed in 2D cultured cells. **B2:** Corresponding trend in 3D spheroid cultures. **(C)** Topographical visualization of sample cells using Z-axis height and deflection data. AFM images were acquired over a 100 × 100 µm² area. **C1: (a)** 2D height map acquired from Z-axis scan (scan forward direction); **(b)** 3D reconstructed surface based on Z-axis scan data; **(c)** Height profile extracted along the scan axis, where the gray line represents raw surface measurements, and the black line shows the mean-fitted curve for smoother interpretation. **C2: (a)** 2D deflection image illustrating surface features based on cantilever bending; **(b)** 3D reconstructed deflection surface; **(c)** Deflection profile along the scan axis, with the gray line indicating raw deflection signals and the black line corresponding to the fitted curve. ‘Epi’ indicates epinephrine, and ‘HC’ indicates hydrocortisone.

Cells mechanical properties can reflect a cell’s potential for migration [39]. Therefore, assessing cell stiffness can provide insight into how these treatments influence the invasive behavior of U87-MG cells in both 2D and 3D cultures (Fig 2A1 and 2A2). Cell stiffness is an important mechanical property. Among the available techniques, atomic force microscopy (AFM) is the most widely used method for measuring stiffness through a quantitative parameter known as Young’s modulus. This modulus is often considered a biomarker for cell motility [40].

A general examination of Fig 2B1 and 2B2 reveals that both epinephrine and hydrocortisone treatments significantly alter the Young’s modulus values of the cells.

**Fig 3:**
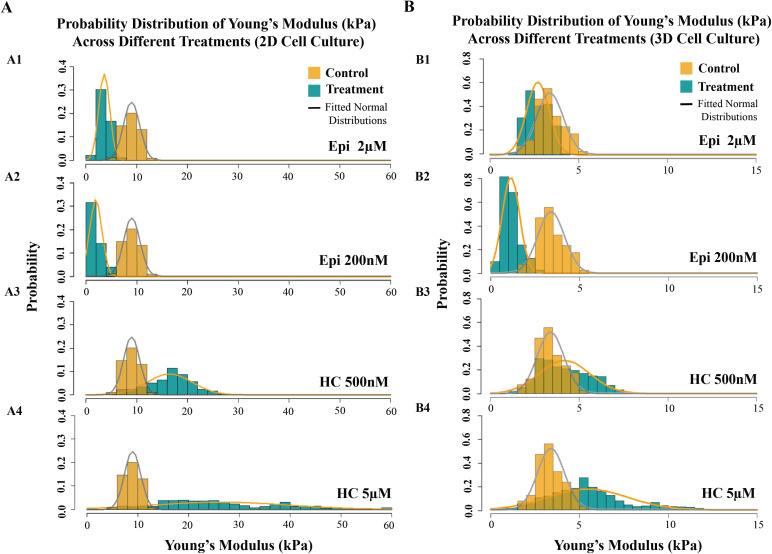
Changes in cell stiffness after treatment in 2D and 3D culture systems. Probability distribution of Young’s Modulus (kPa) for each treatment. Control cells are shown in orange and treated cells in teal. Fitted normal distribution curves are overlaid. **(A)** 2D and **(B)** 3D cell cultures. Panels A1–A4 and B1–B4 correspond to treatments with epinephrine (2 µM and 200 nM) and hydrocortisone (500 nM and 5 µM), respectively. ‘Epi’ indicates epinephrine, and ‘HC’ indicates hydrocortisone.

Fig 2C1 and 2C2 present the height map alongside the reconstructed deflection image, providing a clearer understanding of how AFM scans and visualizes cells. Two important considerations should be noted:

First, as expected, measurements in 3D cultures exhibit greater heterogeneity, likely reflecting the more complex mechanical microenvironment and cell–cell interactions. Second, the overall range of Young’s modulus values varies between 2D (0–60 kPa) and 3D (0–15 kPa) cultures. This variation can be attributed to differences in substrate rigidity — rigid coverslips in 2D versus soft multicellular aggregates in 3D — and the spatial organization of cells Fig 3 [41].

As shown in Fig 2A1–2A2 and 2B1–2B2, epinephrine treatment (at both 2 µM and 200 nM) shifts the Young’s modulus distribution toward lower values in both culture models, indicating a softening of the cells. Interestingly, the lower concentration (200 nM) results in a greater shift, with the distribution peak appearing at smaller modulus values.

Hydrocortisone was also tested at two concentrations (500 nM and 5 µM) in both 2D and 3D cultures. This treatment induces an overall shift toward higher Young’s modulus values (Fig 2A3, 2A4, 2B3, and 2B4), although this shift is more evident in the 2D model. The higher concentration (5 µM) induced a slightly stronger effect. Compared to epinephrine treatment, cells exposed to hydrocortisone appear to exhibit greater heterogeneity in their mechanical responses (Compare Fig 2A1 and 2B1).

**Fig 4:**
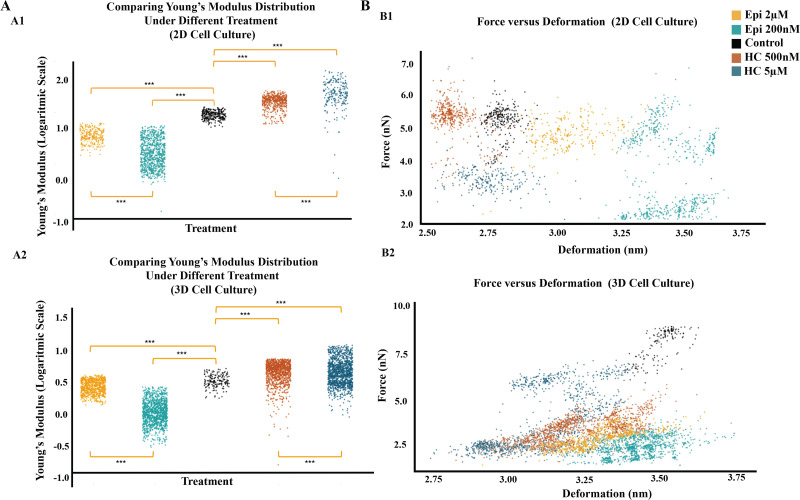
Changes in cell stiffness and deformation profiles following treatment in 2D and 3D cell cultures. **(A)** Comparison of Young’s Modulus values (kPa) across treatment groups; each dot represents an individual measurement. Statistical significance is indicated (***p < 0.001). **(B)** Force versus deformation scatter plots, with each treatment group color-coded as indicated.

Statistical comparisons between treated and control groups, as well as between the two concentration levels, were significant for both treatments (p < 0.0001; Fig 4A1 and 4A2).

Tip force versus cell deformation is shown in Fig 4B1 and 4B2 to illustrate how cells respond to similar forces under epinephrine or hydrocortisone treatment. Although some variability exists, clear trends can be observed in several regions. The same amount of force results in less deformation in hydrocortisone-treated cells and greater deformation in epinephrine-treated cells (Fig 4B1, particularly around the 5 nN axis). Similarly, the same degree of deformation requires less force in epinephrine-treated cells and more force in hydrocortisone-treated cells (Fig 4B2, particularly around the 3.25 nm axis). The inconsistencies may be explained by studies suggesting that, in some cases, effective migration also requires a degree of resistance to deformation [39].

### Both epinephrine and hydrocortisone upregulate vimentin expression

Vimentin is a key cytoskeletal protein involved in epithelial-to-mesenchymal transition (EMT) [33]. Previous studies have suggested that increased vimentin expression may indicate glioblastoma cells increased invasion potential [42,43]. To assess this, vimentin levels were evaluated using immunostaining, analyzed by both flow cytometry and fluorescence microscopy.

**Fig 5:**
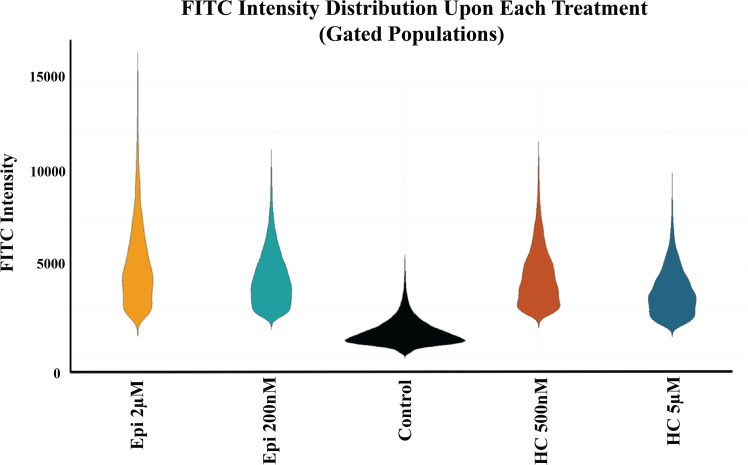
FITC intensity distribution upon each treatment. Violin plots depicting the distribution of vimentin-associated FITC fluorescence intensity in gated cell populations for each treatment group. Higher intensity values indicate increased vimentin expression. Each distribution reflects the heterogeneity of response within treatments. Epi stands for epinephrine and HC stands for hydrocortisone.

**Fig 6:**
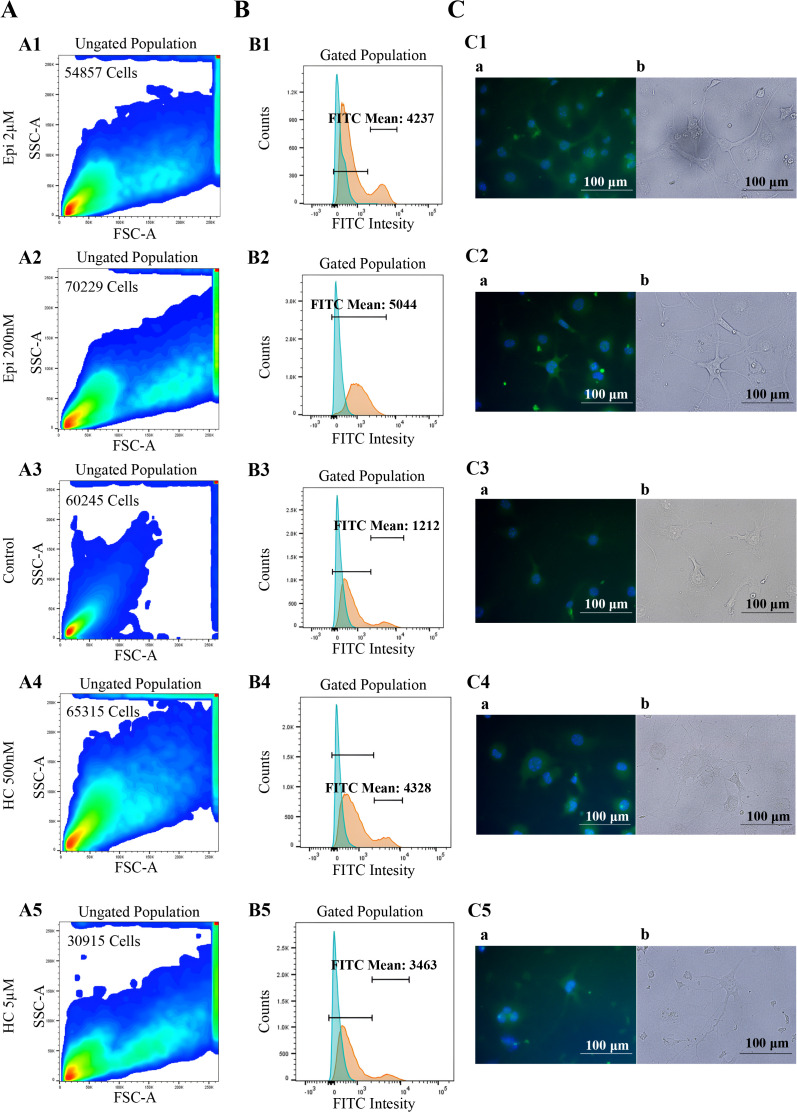
Microscopy and flow cytometry analysis of vimentin expression under different treatment conditions. Rows 1–5 correspond to treatment groups:1: Epinephrine (2 μM), 2: Epinephrine (200 nM), 3: Control (untreated), 4: Hydrocortisone (500 nM), 5: Hydrocortisone (5 μM). Panels **A1–A5**: Flow cytometry density plots showing side scatter area (SSC-A) versus forward scatter area (FSC-A) for the ungated cell populations. Cell density is color-coded, with red indicating the highest and dark blue the lowest density. Panels **B1–B5**: Histograms of FITC fluorescence intensity in gated cell populations. Blue curves represent background fluorescence from unstained controls; orange curves represent cells stained with FITC-conjugated anti-vimentin antibody. Mean fluorescence intensity (MFI) values are shown for the stained populations. Panels **C1–C5**: Immunofluorescence microscopy images. **(a)** Fluorescent images: nuclei stained with DAPI (blue), vimentin with FITC (green); **(b)** Corresponding brightfield images. Scale bars = 100 μm.

As illustrated in Figs 5 and 6A,6B, vimentin expression increased following treatment with both epinephrine and hydrocortisone at both tested concentrations. Notably, the increase was particularly pronounced with 200 nM epinephrine, suggesting highlighted sensitivity at the lower dose.

The gating strategy is detailed in S4A–S4D Fig. In parallel with flow cytometry, fluorescence (Fig 6C1–5) microscopy was used to better visualize changes in vimentin expression.

To assess the statistical significance of these expression changes, the Kolmogorov–Smirnov test was performed on the entire gated cell populations. As shown in Table 1, the distribution of FITC fluorescence intensity differed significantly between control and all treated groups (p < 0.0001). Furthermore, the significant difference observed between the two concentrations of each treatment suggests a dose-dependent effect of vimentin expression. To further support our findings on EMT, we also assessed CD44 expression as another EMT marker. This experiment was conducted once as a confirmatory analysis to complement our vimentin results. As shown in S5 Fig the results were consistent with the vimentin findings.

**Table 1:** Statistical comparison of FITC-A intensity distributions using the Kolmogorov–Smirnov test.

Comparison	D-Statistic	P-Value
Epinephrine 2 µM versus Epinephrine 200 nM	0.15122	<2.2e-16
Control versus Epinephrine 2 µM	0.88419	<2.2e-16
Control versus Epinephrine 200 nM	0.8867	<2.2e-16
Control versus Hydrocortisone 500 nM	0.90877	<2.2e-16
Control versus Hydrocortisone 5 µM	0.77795	<2.2e-16
Hydrocortisone 5 µM versus Hydrocortisone 500 nM	0.21373	<2.2e-16

Each row presents a pairwise comparison between two treatment conditions using the two-sample Kolmogorov–Smirnov test. The D-statistic quantifies the maximum difference between the cumulative distributions, and the p-values indicate statistical significance. All comparisons yielded highly significant differences (p < 2.2 × 10 ⁻ ¹⁶), suggesting that treatment conditions significantly affected the distribution of FITC-A intensities.

## Discussion

In this section, we take a step back to examine how our findings align with the broader body of research regarding the influence of catecholamines and glucocorticoids on glioblastoma invasiveness. Given that the literature encompasses various members of these molecular families and multiple glioblastoma cell lines with distinct genetic backgrounds, we adopt a broad perspective. The studies in this field vary in their objectives, ranging from modeling stress to investigating the therapeutic effects or side effects of these compounds. In this discussion, we primarily focus on *in vitro* studies, as *in vivo*, *ex vivo*, and clinical studies often involve additional variables, such as immune system interactions, that fall outside the scope of our current investigation.

Our findings indicate that epinephrine (200 nM and 2 µM) enhances the migratory behavior of U87-MG cells in both 2D and 3D culture systems. Turning to hydrocortisone, it appears to significantly reduce cell migration across both models at 5 µM, while the lower dose of 500 nM shows no significant effect compared to the control.

These results broadly align with the existing literature. For example, norepinephrine has been shown to enhance migration in U251 and LN229 glioma cell lines [44]. Additionally, one *in vivo* study reported that norepinephrine increases the invasiveness of T98G cells [45]. A recent preprint also proposes a dose-dependent effect of epinephrine on U87 cells. Lower, physiologically relevant concentrations act as migration enhancers, while higher, pharmacological doses suppress cells movement [46]. Our data suggests a possible alignment with this interpretation; epinephrine at 200 nM produced a stronger pro-migratory effect than 2 µM across multiple assays, including 3D migration, 2D and 3D AFM measurements, and vimentin expression. However, the literature is not entirely consistent. One study reported that norepinephrine inhibits migration in U87 and U251 cells, although significant effects were only observed at concentrations of 5 µM and above [47].

Dexamethasone, a synthetic glucocorticoid, has been shown to inhibit the migration of several glioma cell lines, including U251, U373, A172, and U87. Although, findings in the C6 cell line have been mixed, with studies reporting both increased and decreased cell migration following dexamethasone treatment [48]. In U87-MG cells specifically, the inhibitory effect appears to be linked to a reduction in MMP-2 (Matrix Metalloproteinase-2) secretion [49]. However, the findings differ markedly when it comes to glioblastoma stem cells (GSCs). For instance, dexamethasone has been found to enhance the invasive behavior of the GSC3 line. Luedi et al. reported an upregulation of invasion-related gene profiles in both GSC3 and GSC6. Their study also emphasizes that the response to dexamethasone may vary depending on the tumor’s origin, whether it stems from a proneural or mesenchymal subtype [50].

In terms of changes to the mechanical properties of the cells, our results indicate that epinephrine reduces the Young’s Modulus, with values falling below 5 kPa in 3D cultures and below 10 kPa in 2D cultures. In contrast, hydrocortisone increases cell stiffness, reaching approximately 60 kPa in 2D cultures and about 12 kPa in 3D cultures. A wide range of Young’s Modulus values has been reported for glioblastoma cell lines, ranging from 0.08 to 119 kPa [51]. However, it is important to note that in our study, the cells were seeded for four days, which may not directly align with these established baselines. As suggested by the survey conducted by Luo et al., [40] several factors, such as the cantilever and tip used in AFM, can influence the results. Therefore, it is more meaningful to interpret our findings in the context of internal comparisons within this study rather than comparing them to baseline values.

While the effects of molecular stress modulators on stiffness have not yet been fully explored, existing evidence indicates changes to the cytoskeleton that align with our findings. For example, a preprint suggests that epinephrine reduces U87 cell adhesion [52]. More information is available regarding glucocorticoids. Studies show that dexamethasone treatment induces actin reorganization in glioblastoma cells, shifting from a cortical arrangement to a more prominent stress fiber configuration. It also activates fibronectin matrix assembly. These cytoskeletal and adhesion changes promote a transition from a rounder to a flatter, more adherent cell morphology, contributing to increased stiffness. The increase in cell-cell and cell-substrate adhesion also corresponds with our findings in 3D migration assays, where hydrocortisone treatment caused a reduction in the cells’ dispersal velocity [53,54].

Overall, our data on migration and AFM indicate that lower cell stiffness is associated with increased migratory capacity, while higher stiffness corresponds to reduced migration [40].

Our final set of results focused on changes in EMT-molecular markers. We selected vimentin for this study because it is a well-established marker of EMT and is closely associated with cell motility in gliomas [55,56]. Based on our findings from the other assays, we expected epinephrine would increase vimentin expression, while hydrocortisone would decrease it. However, our results indicated that both treatments led to an increase in vimentin levels.

For epinephrine, this outcome aligns with vimentin’s role in promoting EMT and enhanced cellular motility. In the case of hydrocortisone, we propose that the increase in vimentin is linked to the induction of stemness features, as vimentin is associated with stem cell-like phenotypes [57]. Previous studies have demonstrated that dexamethasone can promote a stem cell-like state, which may help explain our findings [58]. Interestingly, other research indicates that increased vimentin expression occurs in response to anticancer drugs, suggesting that EMT may not only signal cancer progression but also reflect a potential mechanism [59]. One other explanation is that vimentin up-regulation may reflect distinct organizational states of the intermediate filament network, leading to different cell stiffness [60].

As a complementary assay following vimentin results, CD44 expression was analyzed for both epinephrine and hydrocortisone treatments. CD44 is a cell surface glycoprotein involved in cell adhesion and migration, and its expression has been well characterized in U87 glioblastoma cells [61].

In our study, the proportion of CD44 ⁺ cells increased after both treatments, while the mean fluorescence intensity (MFI) remained relatively constant across conditions. This pattern suggests a population-level shift toward a more mesenchymal or stem-like phenotype. Both epinephrine and hydrocortisone may induce expansion of a preexisting CD44 ⁺ subpopulation. There are several possible explanations for this observation. First, CD44 ⁺ cells are often more resistant to cytotoxic agents [62], so their proliferation and survival may be less compromised during mitomycin c treatment [62]. Second, the treatments may selectively promote survival or proliferation of CD44 ⁺ cells over CD44 ⁻ cells, effectively enriching this subpopulation. Third, the observed increase in CD44 ⁺ cells may reflect a treatment-dependent shift in isoform expression, with different CD44 variant isoforms becoming more prevalent depending on the stimulus. Some of these variants are associated with either migratory or adhesive phenotypes. This is consistent with previous reports demonstrating that CD44 isoforms can differentially regulate cell adhesion, migration, and stem-like properties [63].Although mitomycin c was applied for a short period (2–3 hours) and removed before the primary stress modulator treatments, the possibility of residual cellular stress cannot be completely ruled out. Nevertheless, mitomycin c exposure at such a short duration is unlikely to exert strong, lasting transcriptional effects, particularly since comet assay results confirmed the absence of significant DNA damage.

For future studies, it would be valuable to more clearly differentiate between cell migration and invasion, ideally using scaffold-based 3D culture systems that more accurately replicate the tumor microenvironment. This approach allows us to directly study cell capability to degrade ECM. Additionally, expanding the panel of EMT markers beyond vimentin and CD44 could provide a more comprehensive understanding of the phenotypic changes induced by treatment. Finally, further research is necessary to elucidate the complex relationship between EMT and stemness.

### Conclusion

Our findings suggest that epinephrine enhances the invasive potential of U87-MG cells, accompanied by a reduction in cell stiffness that is likely facilitating migration. In contrast, hydrocortisone suppresses invasion, resulting in a corresponding increase in stiffness that aligns with a reduced migratory capacity. Both treatments elevated vimentin levels, but likely for different reasons. While epinephrine appears to trigger EMT, hydrocortisone may increase vimentin as part of a shift toward a stem-like phenotype.

## Supporting information

S1 Fig U87-MG cells in 2D and 3D cell culture. To further examine the consistency of results across different culture methods, both 2D and 3D cell culture systems were established. S1 Fig A and B confirm the successful formation of monolayer and spheroid cultures, respectively, providing a basis for subsequent comparative analyses.
(PDF)

S2 Fig Assessment of DNA damage by comet assay in U87-MG cells following Mitomycin C treatment. Panels A and B show comet assay results at low magnification (scale bar: 100 µm), while panels C and D display representative nuclei at higher magnification (scale bar: 30 µm). Panel C shows the positive control (5% H₂O₂) with pronounced comet tails, indicating DNA fragmentation. Panels A and D show mitomycin C-treated cells, which exhibit compact nuclei with minimal or no tail formation, suggesting effective cell cycle arrest without substantial DNA damage. Panel B shows the negative control (untreated cells) with intact nuclei and no detectable DNA fragmentation.
(PDF)

S3 Fig Flow cytometry analysis confirming G2/M arrest following mitomycin c treatment. U87-MG cells were stained with propidium iodide and analyzed by flow cytometry to assess cell cycle distribution. (A) In control cells, 8.9% of the population was in the G2 phase. (B) Treatment with mitomycin c resulted in an increase to 18.3% G2-phase cells, confirming G2/M arrest. Peaks correspond to different phases of the cell cycle, with the G2 region marked in blue, the G1 phase in orange, and the S phase in green.
(PDF)

S4 Fig Gating strategy for flow cytometry analysis and doublet discrimination. (A) Forward scatter area (FSC-A) versus side scatter area (SSC-A) plot used to gate the main cell population based on size and granularity. (B) FSC-A versus forward scatter height (FSC-H) plot used to exclude doublets based on signal height. (C) FSC-A versus forward scatter width (FSC-W) plot used to further exclude doublets based on signal width. (D) Schematic illustration of flow cytometry signal parameters for singlet versus doublet discrimination, showing how signal height, width, and area differ between singlets and doublets.
(PDF)

S5 Fig Both epinephrine and hydrocortisone increase the proportion of CD44 ⁺ cells. Histograms show APC fluorescence intensity in gated cell populations. Panels A1–A5 correspond to the treatment groups: 1, Control (untreated); 2, Epinephrine (2 μM); 3, Epinephrine (200 nM); 4, Hydrocortisone (500 nM); 5, Hydrocortisone (5 μM). The orange-shaded area indicates the CD44 ⁺ cell population.
(PDF)

S1 File Graphical abstract. (DOCX)
