# Supplementary material for: A comparative investigation of catecholamines and glucocorticoids impact on glioblastoma invasive behavior via 2D and 3D cell culture
Source: PLoS One. 2026 Feb 11;21(2):e0339764. doi: 10.1371/journal.pone.0339764 (PMC12893578; doi:10.1371/journal.pone.0339764)
Supplement: S1 Fig — To further examine the consistency of results across different culture methods, both 2D and 3D cell culture systems were established. S1 Fig A and B confirm the successful formation of monolayer and spheroid cultures, respectively, providing a basis for subsequent comparative analyses. (PDF) [file pone.0339764.s001.pdf]

Supporting Information

1. 2D and 3D cell culture

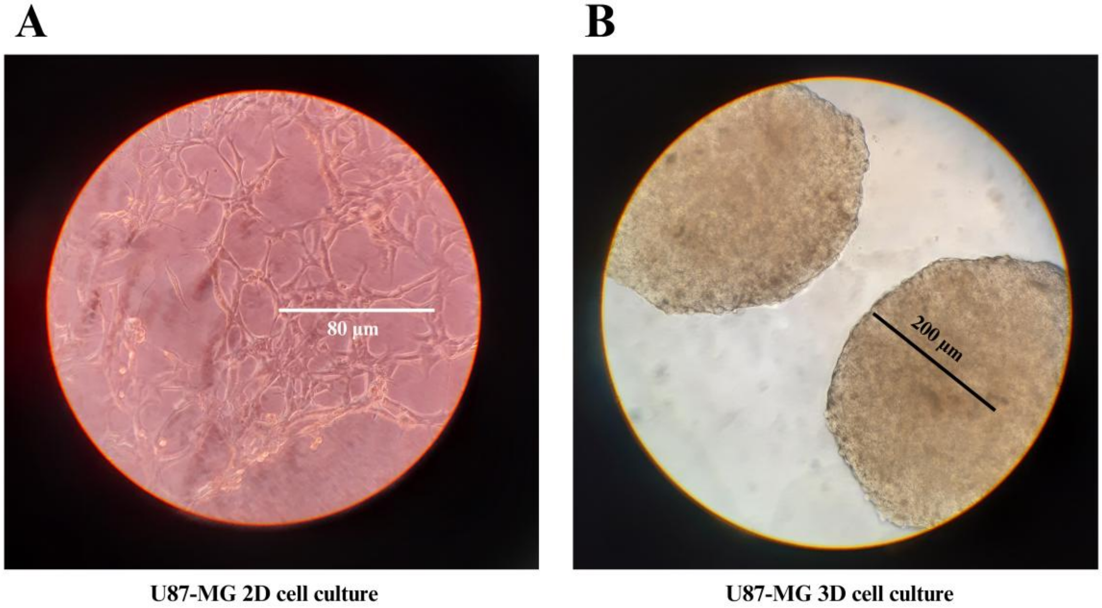

S1 Fig. U87-MG cells in 2D and 3D cell culture. (a) U87-MG cells in 2D monolayer. (b) U87-MG spheroids.

To further examine the consistency of results across different culture methods, both 2D and 3D cell culture systems were established. S1 Fig A and B confirm the successful formation of monolayer and spheroid cultures, respectively, providing a basis for subsequent comparative analyses.
