# Supplementary material for: A comparative investigation of catecholamines and glucocorticoids impact on glioblastoma invasive behavior via 2D and 3D cell culture
Source: PLoS One. 2026 Feb 11;21(2):e0339764. doi: 10.1371/journal.pone.0339764 (PMC12893578; doi:10.1371/journal.pone.0339764)
Supplement: S2 Fig — Panels A and B show comet assay results at low magnification (scale bar: 100 µm), while panels C and D display representative nuclei at higher magnification (scale bar: 30 µm). Panel C shows the positive control (5% H₂O₂) with pronounced comet tails, indicating DNA fragmentation. Panels A and D show mitomycin C-treated cells, which exhibit compact nuclei with minimal or no tail formation, suggesting effective cell cycle arrest without substantial DNA damage. Panel B shows the negative control (untreated cells) with intact nuclei and no detectable DNA fragmentation. (PDF) [file pone.0339764.s002.pdf]

2. Comet assay after low-dose, short-term mitomycin c treatment

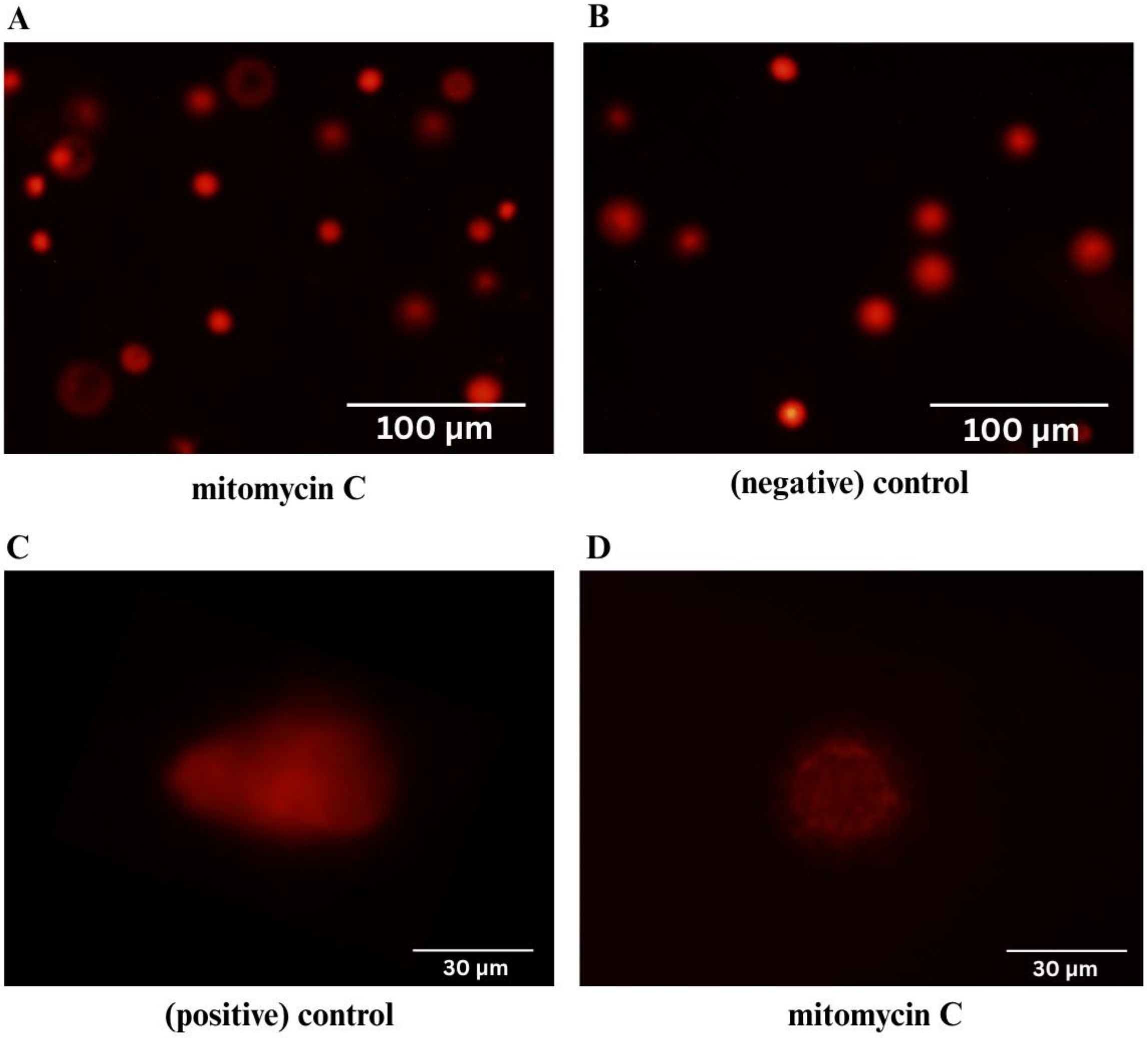

S2 Fig. Assessment of DNA damage by comet assay in U87-MG cells following Mitomycin C treatment. Panels A and B show comet assay results at low magnification (scale bar: 100 µm), while panels C and D display representative nuclei at higher magnification (scale bar: 30 µm). Panel C shows the positive control (5% H_2O_2) with pronounced comet tails, indicating DNA fragmentation. Panels A and D show mitomycin C-treated cells, which exhibit compact nuclei with minimal or no tail formation, suggesting effective cell cycle arrest without substantial DNA damage. Panel B shows the negative control (untreated cells) with intact nuclei and no detectable DNA fragmentation.

Mitomycin c arrests the cell cycle at the G2/M phase by interfering with DNA replication through the formation of DNA crosslinks and inducing DNA damage. Since DNA damage has been shown to promote invasive behavior in cancer cells, it was important to ensure that Mitomycin C treatment did not cause significant DNA fragmentation before administering hydrocortisone and epinephrine. To assess this, a comet assay was performed. As shown in S2 Fig B and E, cells treated with Mitomycin C displayed no visible DNA fragmentation, similar to the negative control (S2 Fig C), where the DNA remains compact within the nucleus. In contrast, the positive control (S2 Fig A and D) exhibited clear tail formation, indicating DNA damage and confirming the validity of the assay.
