# Supplementary material for: A comparative investigation of catecholamines and glucocorticoids impact on glioblastoma invasive behavior via 2D and 3D cell culture
Source: PLoS One. 2026 Feb 11;21(2):e0339764. doi: 10.1371/journal.pone.0339764 (PMC12893578; doi:10.1371/journal.pone.0339764)
Supplement: S3 Fig — U87-MG cells were stained with propidium iodide and analyzed by flow cytometry to assess cell cycle distribution. (A) In control cells, 8.9% of the population was in the G2 phase. (B) Treatment with mitomycin c resulted in an increase to 18.3% G2-phase cells, confirming G2/M arrest. Peaks correspond to different phases of the cell cycle, with the G2 region marked in blue, the G1 phase in orange, and the S phase in green. (PDF) [file pone.0339764.s003.pdf]

3. Flow cytometry analysis to confirm cell cycle arrest induced by mitomycin c

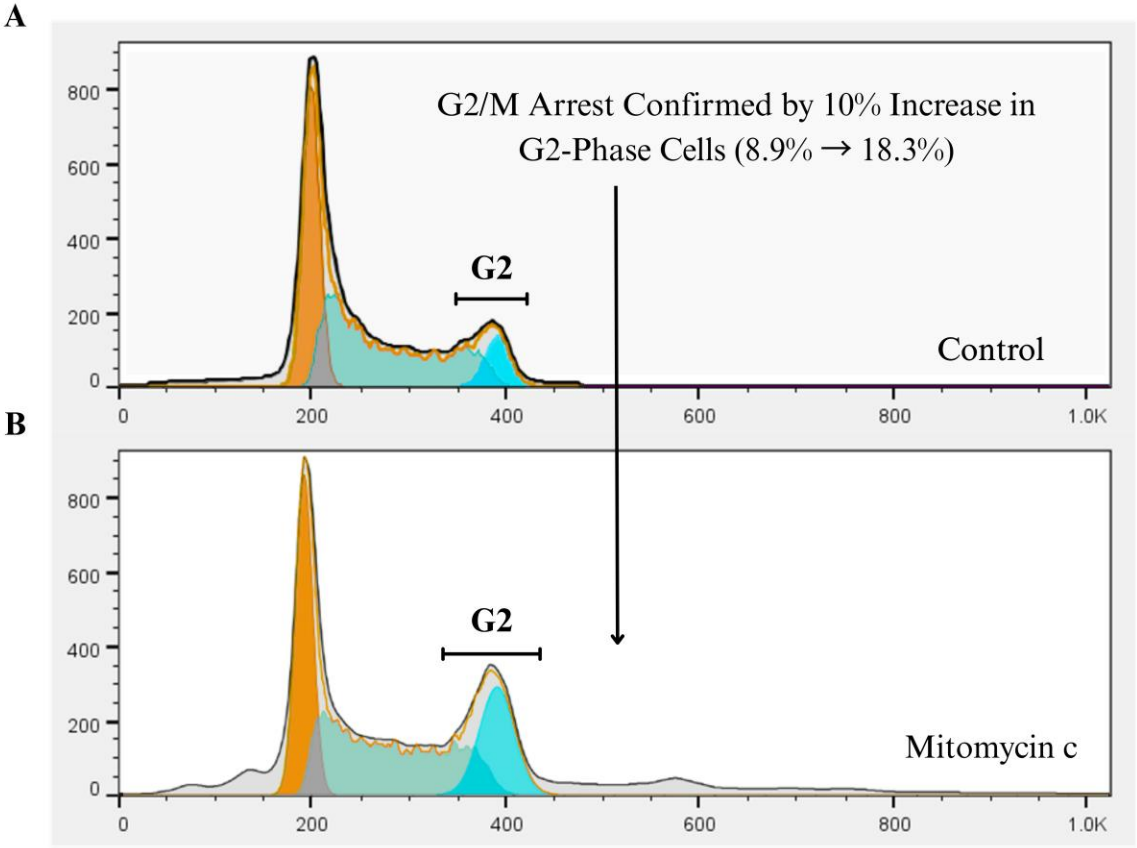

S3 Fig. Flow cytometry analysis confirming G2/M arrest following mitomycin c treatment. U87-MG cells were stained with propidium iodide and analyzed by flow cytometry to assess cell cycle distribution. (A) In control cells, 8.9% of the population was in the G2 phase. (B) Treatment with mitomycin c resulted in an increase to 18.3% G2-phase cells, confirming G2/M arrest. Peaks correspond to different phases of the cell cycle, with the G2 region marked in blue, the G1 phase in orange, and the S phase in green.

To maintain cells within a single generation mitomycin c was used to arrest the cell cycle before applying epinephrine and hydrocortisone treatments. This allowed us to assess the impact of stress-related modulators specifically on cell invasion. Cell cycle arrest was confirmed by flow cytometry. As shown in S3 Fig B, mitomycin c treated cells exhibited an increased proportion of G2-phase cells compared to the control group in S3 Fig A.
