# Supplementary material for: A comparative investigation of catecholamines and glucocorticoids impact on glioblastoma invasive behavior via 2D and 3D cell culture
Source: PLoS One. 2026 Feb 11;21(2):e0339764. doi: 10.1371/journal.pone.0339764 (PMC12893578; doi:10.1371/journal.pone.0339764)
Supplement: S4 Fig — (A) Forward scatter area (FSC-A) versus side scatter area (SSC-A) plot used to gate the main cell population based on size and granularity. (B) FSC-A versus forward scatter height (FSC-H) plot used to exclude doublets based on signal height. (C) FSC-A versus forward scatter width (FSC-W) plot used to further exclude doublets based on signal width. (D) Schematic illustration of flow cytometry signal parameters for singlet versus doublet discrimination, showing how signal height, width, and area differ between singlets and doublets. (PDF) [file pone.0339764.s004.pdf]

4. Cell population gating approach for precise analysis of vimentin expression.

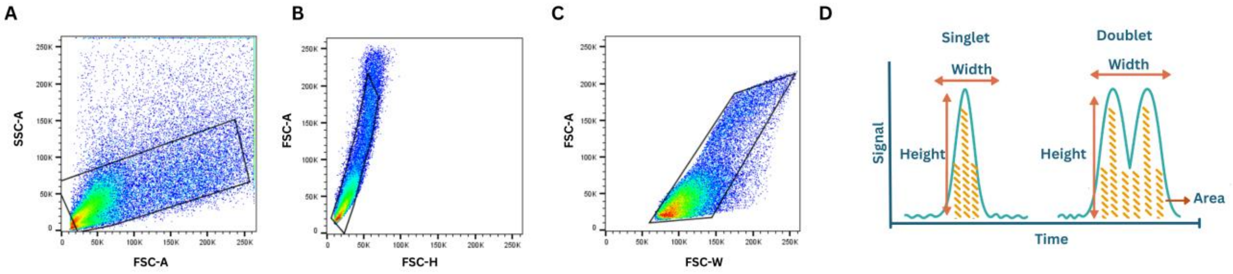

S4 Fig. Gating strategy for flow cytometry analysis and doublet discrimination. (A) Forward scatter area (FSC-A) versus side scatter area (SSC-A) plot used to gate the main cell population based on size and granularity. **(B)** FSC-A versus forward scatter height (FSC-H) plot used to exclude doublets based on signal height. **(C)** FSC-A versus forward scatter width (FSC-W) plot used to further exclude doublets based on signal width. **(D)** Schematic illustration of flow cytometry signal parameters for singlet versus doublet discrimination, showing how signal height, width, and area differ between singlets and doublets.
