# Supplementary material for: A comparative investigation of catecholamines and glucocorticoids impact on glioblastoma invasive behavior via 2D and 3D cell culture
Source: PLoS One. 2026 Feb 11;21(2):e0339764. doi: 10.1371/journal.pone.0339764 (PMC12893578; doi:10.1371/journal.pone.0339764)
Supplement: S5 Fig — Histograms show APC fluorescence intensity in gated cell populations. Panels A1–A5 correspond to the treatment groups: 1, Control (untreated); 2, Epinephrine (2 μM); 3, Epinephrine (200 nM); 4, Hydrocortisone (500 nM); 5, Hydrocortisone (5 μM). The orange-shaded area indicates the CD44 ⁺ cell population. (PDF) [file pone.0339764.s005.pdf]

5. Both epinephrine and hydrocortisone increase the proportion of $CD44^{+}$ cells

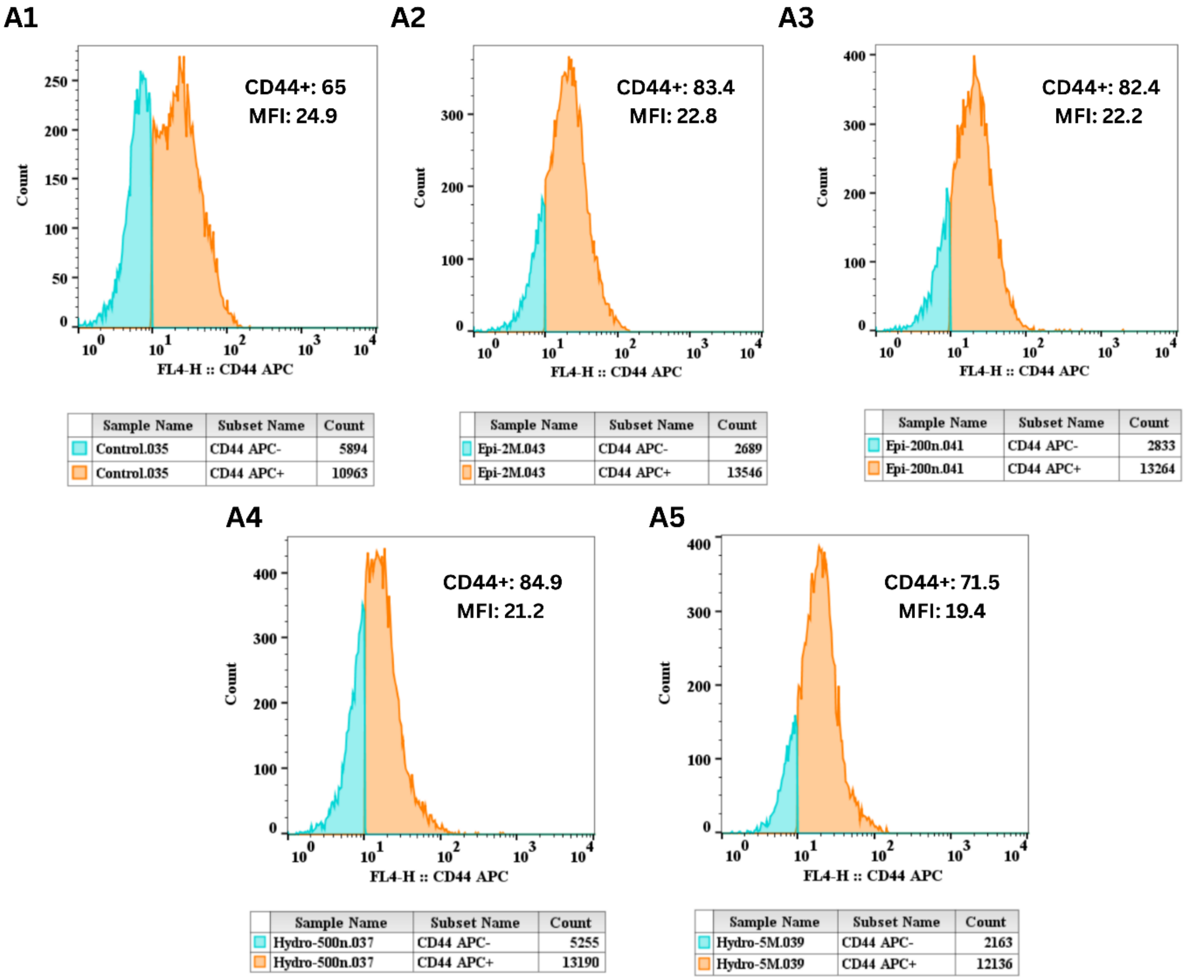

S5 Fig. Both epinephrine and hydrocortisone increase the proportion of $CD44^{+}$ cells. Histograms show APC fluorescence intensity in gated cell populations. Panels A1–A5 correspond to the treatment groups: 1, Control (untreated); 2, Epinephrine (2 µM); 3, Epinephrine (200 nM); 4, Hydrocortisone (500 nM); 5, Hydrocortisone (5 µM). The orange-shaded area indicates the $CD44^{+}$ cell population.
