# Supplementary material for: A comparative investigation of catecholamines and glucocorticoids impact on glioblastoma invasive behavior via 2D and 3D cell culture
Source: PLoS One. 2026 Feb 11;21(2):e0339764. doi: 10.1371/journal.pone.0339764 (PMC12893578; doi:10.1371/journal.pone.0339764)
Supplement: S1 File — (DOCX) [file pone.0339764.s006.docx]

Graphical Abstract

This study investigates the impact of molecular stress modulators on the invasiveness of glioblastoma. U87-MG glioblastoma cells were treated with epinephrine (Epi) or hydrocortisone (HC) in both 2D and 3D cultures. Epinephrine promoted cell migration, reduced cellular stiffness, and increased vimentin expression, indicating enhanced invasiveness driven by EMT. In contrast, hydrocortisone suppressed migration, increased stiffness, and elevated vimentin levels, likely through a mechanism unrelated to EMT. These findings illustrate how different stress responses can distinctly influence tumor cell behavior and mechanical properties.
